# Endothelin-1 in Paraventricular Nucleus Modulates Cardiac Sympathetic Afferent Reflex and Sympathetic Activity in Rats

**DOI:** 10.1371/journal.pone.0040748

**Published:** 2012-07-16

**Authors:** Ai-Dong Chen, Xiao-Qing Xiong, Xian-Bing Gan, Feng Zhang, Ye-Bo Zhou, Xing-Ya Gao, Ying Han

**Affiliations:** 1 Department of Physiology, Nanjing Medical University, Nanjing, China; 2 Department of Clinical Pharmacy, Yijishan Hospital of Wannan Medical College, Wuhu, China; Max-Delbrück Center for Molecular Medicine (MDC), Germany

## Abstract

**Background:**

Cardiac sympathetic afferent reflex (CSAR) is a positive-feedback, sympathoexcitatory reflex. Paraventricular nucleus (PVN) is an important component of the central neurocircuitry of the CSAR. The present study is designed to determine whether endothelin-1 (ET-1) in the PVN modulates the CSAR and sympathetic activity, and whether superoxide anions are involved in modulating the effects of ET-1 in the PVN in rats.

**Methodology/Principal Findings:**

In anaesthetized Sprague–Dawley rats with cervical vagotomy and sinoaortic denervation, renal sympathetic nerve activity (RSNA) and mean arterial pressure (MAP) were recorded. The CSAR was evaluated by the responses of the RSNA and MAP to epicardial application of capsaicin. Microinjection of ET-1 into the bilateral PVN dose-dependently enhanced the CSAR, increased the baseline RSNA and MAP. The effects of ET-1 were blocked by PVN pretreatment with the ET_A_ receptor antagonist BQ-123. However, BQ-123 alone had no significant effects on the CSAR, the baseline RSNA and MAP. Bilateral PVN pretreatment with either superoxide anion scavenger tempol or polyethylene glycol-superoxide dismutase (PEG-SOD) inhibited the effects of ET-1 on the CSAR, RSNA and MAP. Microinjection of ET-1 into the PVN increased the superoxide anion level in the PVN, which was abolished by PVN pretreatment with BQ-123. Epicardial application of capsaicin increased superoxide anion level in PVN which was further enhanced by PVN pretreatment with ET-1.

**Conclusions:**

Exogenous activation of ET_A_ receptors with ET-1 in the PVN enhances the CSAR, increases RSNA and MAP. Superoxide anions in PVN are involved in the effects of ET-1 in the PVN.

## Introduction

Endothelins (ET) are now recognized for their function in central regulation of cardiovascular activity [1], [2]. In central nervous system (CNS), ET-1 is produced by vascular endothelial cells, neurone and/or non-neural elements or delivered by way of the cerebrospinal fluid [3].It has been reported that injection of ET-1 into the cerebral ventricles evokes a pressor response in rats [4], which is mediated by increased sympathetic outflow [5]. Intracerebroventricular administration of the ET_A_ receptor antagonist BQ-123 abolishes the pressor response to ET-1 [6]. Intracerebroventricular injections of ET-1 increases sympathetic nerve activity and blood pressure via ET_A_ receptors but not via ET_B_ receptors in rats [7]. These results suggest that central ET-1 is involved in the modulation of blood pressure and sympathetic output. Earlier studies have shown that the pressor response induced by central ET-1 is due to stimulation of arginine vasopressin (AVP) release [4], [8]. However, systemic vasopressin receptor blockade does not inhibit the pressor response induced by central ET-1, and arterial pressure rises equally in normotensive Long Evans rats and in the Brattleboro strain devoid of central AVP [9]. Furthermore, the response of ET-1 in the baroreflex-intact rats is independent from the AVP release [1]. These results suggest that the AVP is not the necessary factor in the pressor response to central ET-1. The mechanisms involved in the pressor and sympathoexcitatory responses to ET-1 in the CNS are still unknown.

It is well established that cardiac sympathetic afferent reflex (CSAR) is a positive-feedback, sympathoexcitatory reflex. The enhanced CSAR partially contributes to the sympathetic excitation in the chronic heart failure (CHF) state [10]–[12] and hypertension [13], [14]. Paraventricular nucleus (PVN) is an important integrative center in the control of the CSAR [15], [16]. Abundant ET-1 expression is found in the PVN [17], especially in the parvocellular PVN cells [18]. Autoradiographic visualization of the binding sites for ^[125I]^endothelin shows both ET_A_ receptors and ET_B_ receptor expression in the PVN [3], [19], [20].The lesion of the PVN prevents the intracerebroventricular administration of ET-1-induced increase in arterial pressure [6]. The aminopropionic acid receptors in the PVN are involved in mediating the pressor response to ET-1 in subfornical organ (SFO) [21]. These results suggest that PVN plays an important role in the central effects of ET-1 and ET-1 and ET_A_ receptors in the PVN may be involved in regulating the CSAR which contributes to the sympathetic excitation.

Previous studies in our lab have shown that NAD(P)H oxidase-derived reactive oxygen species (ROS) especially superoxide anions in the PVN mediate the CSAR and contributes to the effect of Ang II in the PVN on the CSAR in rats [22], [23]. The superoxide anions in the PVN mediate the enhanced sympathetic outflow and CSAR in rats with CHF [24] and hypertension [25]. Accumulated evidences indicate that endothelins activate NAD(P)H oxidases and thereby increase superoxide production, resulting in oxidative stress and cardiovascular dysfunction [26]–[29]. The aims of this study were to investigate the role of ET-1 and ET_A_ receptors in the PVN in regulating the CSAR and sympathetic activity and the involvement of the superoxide anions in mediating the effects of ET-1 and ET_A_ in the PVN in rats.

## Materials and Methods

Experiments were carried out in male Sprague-Dawley rats weighing between 300 and 400 g. The procedures were approved by the Experimental Animal Care and Use Committee of Nanjing Medical University (No. 20110115) and complied with the Guide for the Care and Use of Laboratory Animals (NIH Publication No. 85–23, revised 1996).

### General Procedures

The rats were anesthetized with urethane (800 mg/kg, ip) and α-chloralose (40 mg/kg, ip). Adequate depth of anesthesia was assessed by the absence of corneal reflexes and paw withdrawal response to a noxious pinch. Supplemental doses of urethane and α-chloralose were administered as necessary to maintain an adequate depth of anesthesia. The trachea was cannulated for positive-pressure ventilation using a rodent ventilator (51600, Stoelting, USA) with room air. Arterial blood pressure (ABP) was measured with a pressure transducer (MLT0380, ADInstruments, Australia) through a catheter placed into the right carotid artery. Baroreceptor denervation and vagotomy were carried out and identified as previously reported [22]. Body temperature was maintained at 37±1°C with a heating pad.

### PVN Microinjection

Rats were placed in a stereotaxic frame (Stoelting, Chicago). The coordinates for PVN were determined according to the Paxinos and Watson rat atlas [30], which is 1.8 mm caudal from bregma, 0.4 mm lateral to the midline, and 7.9 mm ventral to the dorsal surface. The bilateral PVN microinjections were completed within 1 minute and the microinjection volume for each side was 50 nl. At the end of the experiment, 50 nl of Evans blue (2%) was injected into the microinjection site for the later histological verification. Rats with microinjection sites out of the PVN were excluded from data analysis.

### Renal Sympathetic Nerve Activity (RSNA) Recordings

Renal sympathetic nerve was isolated through a retroperitoneal incision. The renal nerve was cut distally to eliminate its afferent activity. The nerve was then placed on a pair of silver electrodes and was immersed in mineral oil. The nerve signals were amplified with an AC/DC differential amplifier (Model 3000, A-M System Inc.) with a low-frequency cutoff at 60 Hz and a high-frequency cutoff at 3000 Hz. The amplified and filtered signals were integrated at time constant of 10 ms. After the section of the central end of the renal nerve at the end of each experiment, the background noise was determined and was subtracted from the integrated values of the RSNA. The raw RSNA, integrated RSNA, ABP and mean arterial pressure (MAP) were simultaneously recorded on a PowerLab data acquisition system (8SP, ADInstruments, Australia) and stored on hard disk. The RSNA was expressed as the percent change from the baseline value.

### Evaluation of the CSAR

The heart was exposed with a limited left lateral thoracotomy and the pericardium was removed. The CSAR was elicited by application of a piece of filter paper (3×3 mm) containing capsaicin (1.0 nmol in 2.0 µl) to the epicardial surface of anterior wall of the left ventricle [31]. Each piece of paper was removed 1 minute later. The epicardium was rinsed three times with 10 ml of warm normal saline (38°C). The CSAR was evaluated by the responses of the RSNA and MAP to epicardial application of capsaicin.

### Measurement of Superoxide Anion Level

The brain of rat was removed from the skull quickly and flash-frozen in liquid nitrogen and stored at −70°C. Coronal section of the brain was made with cryostat microtome (CM1900, Leica LTD), and the PVN area was punched out with a 15-gauge needle. The punched tissue of the PVN was homogenized and then centrifuged. Protein concentrations in the supernatants were measured with the Bradford assay [32].

Lucigenin-derived chemiluminescence is a valid probe for detecting superoxide anions [33]–[36]. Superoxide anion level was measured with lucigenin-derived chemiluminescence as our previous report [24]. Briefly, the reaction with superoxide anions was started by addition of dark-adapted lucigenin (5 µM) to sample to cause photon emission which was measured with a luminometer (20/20 n, Turner, CA) for ten times. Average values were calculated and expressed as mean light unit (MLU) per minute per milligram of protein, which represented the superoxide anion level. Background chemiluminescence in the buffer that contains lucigenin was measured.

### Drugs

Capsaicin, endothelin-1, BQ123 (cyclo-(D-Asp-Pro-D-Val-Leu-D-Trp), tempol, polyethylene glycol-superoxide dismutase (PEG-SOD) were obtained from Sigma Chemical Co. The drugs were dissolved in artificial cerebrospinal fluid (ACSF) except the capsaicin in normal saline. The ACSF contained the following (mM): 10 dextrose, 125 NaCl, 24 NaHCO_3_, 5 KCl, 2.5 CaCl_2_, 1.25 MgSO_4_, 1.25 KH_2_PO_4_).

### Protocols

Firstly, the effects of PVN microinjection of different doses of ET-1 (0, 0.1, 1 and 10 pmol) on the CSAR, the baseline RSNA and MAP were determined in four groups of rats respectively (n = 6 for each group). Ten minutes after the PVN microinjection, the CSAR was evaluated by the RSNA and MAP responses to epicardial application of capsaicin. To exclude the possibility that the effects of ET-1 on the CSAR were caused by diffusion to other brain area, the effects of microinjection of ET-1 (10 pmol) into the anterior hypothalamic area which is adjacent to the PVN were determined (n = 3).

Secondly, the effects of the selective ET_A_ receptor antagonist, BQ123, in the PVN on the CSAR, the baseline RSNA and MAP and responses induced by ET-1 were determined. PVN microinjection of ACSF, ET-1 (10 pmol), BQ123 (40 nmol) and ET-1 pretreated with BQ123 were carried out in four groups of rats (n = 6 for each group). Ten minutes after the PVN microinjection, the CSAR was evaluated. The PVN pretreatment with BQ123 was carried out 15 minutes before ET-1.

Thirdly, the effects of tempol and PEG-SOD in the PVN on the enhanced CSAR and RSNA responses as well as pressor response induced by ET-1 were investigated. PVN microinjection of ACSF, tempol (20 nmol) and PEG-SOD (2 units) alone, as well as ET-1 (10 pmol) pretreated with ACSF, tempol, and PEG-SOD were carried out in six groups of rats (n = 6 for each group). The pretreatment was carried out 15 minutes before ET-1. The CSAR was determined 10 minutes after the PVN microinjection.

Lastly, the effects of the PVN microinjection of ACSF, ET-1 (10 pmol), BQ123 (40 nmol), and ET-1 pretreated with BQ123 on the superoxide anion level in the PVN were determined in 4 groups of rats (n = 6 for each group). The pretreatment was administered 15 minutes before ET-1. Ten minutes after PVN microinjection, the rat was decapitated and prepared for measurement of superoxide anion level in the PVN.

### Statistics

Comparisons between groups were made by one-way ANOVA followed by the Newman-Keuls test for post hoc analysis. All statistical analyses were done using computer software (SigmaStat, SPSS 10.0). All data were expressed as mean ± SE. *P*<0.05 was considered statistically significant.

## Results

### Effects of Different Doses of ET-1

**Figure 1:**
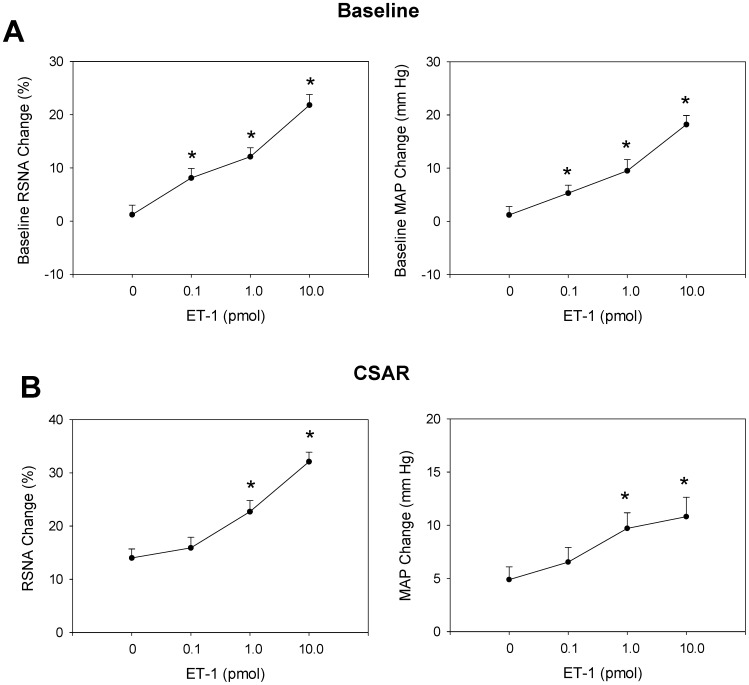
Effects of the PVN microinjection of different doses of ET-1 (0,0.1,1 and 10 pmol) on the baseline RSNA and MAP (A) and CSAR (B). The CSAR was evaluated by the RSNA and MAP responses to epicardial application of capsaicin. Values are mean ± SE. *P<0.05 compared with control. n = 6 for each group.

Microinjection of three doses of ET-1 (0.1, 1, 10 pmol) into the PVN induced dose-related increases in CSAR, baseline RSNA and MAP. Compared with control, three doses of ET-1 significantly increased the baseline RSNA and MAP (Fig. 1A), but only middle and high doses of ET-1 significantly increased the CSAR (Fig. 1B). Baseline MAP before PVN microinjection and after CSAR has been shown in Table 1. However, microinjection of high dose of ET-1 into the anterior hypothalamic area which is adjacent to the PVN failed to cause any significant effect on the CSAR.

**Table 1:** Baseline MAP before PVN microinjection and after epicardial application of capsaicin to induce CSAR.

	Before PVN microinjection	After capsaicin
ACSF	90.4±2.3	96.0±2.9
ET-1 (0.1 pmol)	90.0±3.5	101.9±4.2*
ET-1 (1 pmol)	91.2±4.5	110.4±5.1* ^†^
ET-1 (10 pmol)	90.2±3.0	119.2±2.0* ^†^

Ten minutes after the PVN microinjection, the CSAR was evaluated by the RSNA and MAP responses to epicardial application of capsaicin. Values are expressed as mean±SE.

* *P*<0.05 vs. the data before PVN microinjection, ^†^ *P*<0.05 vs. ACSF. *n = 6* for each group.

### Effects of BQ123

**Figure 2:**
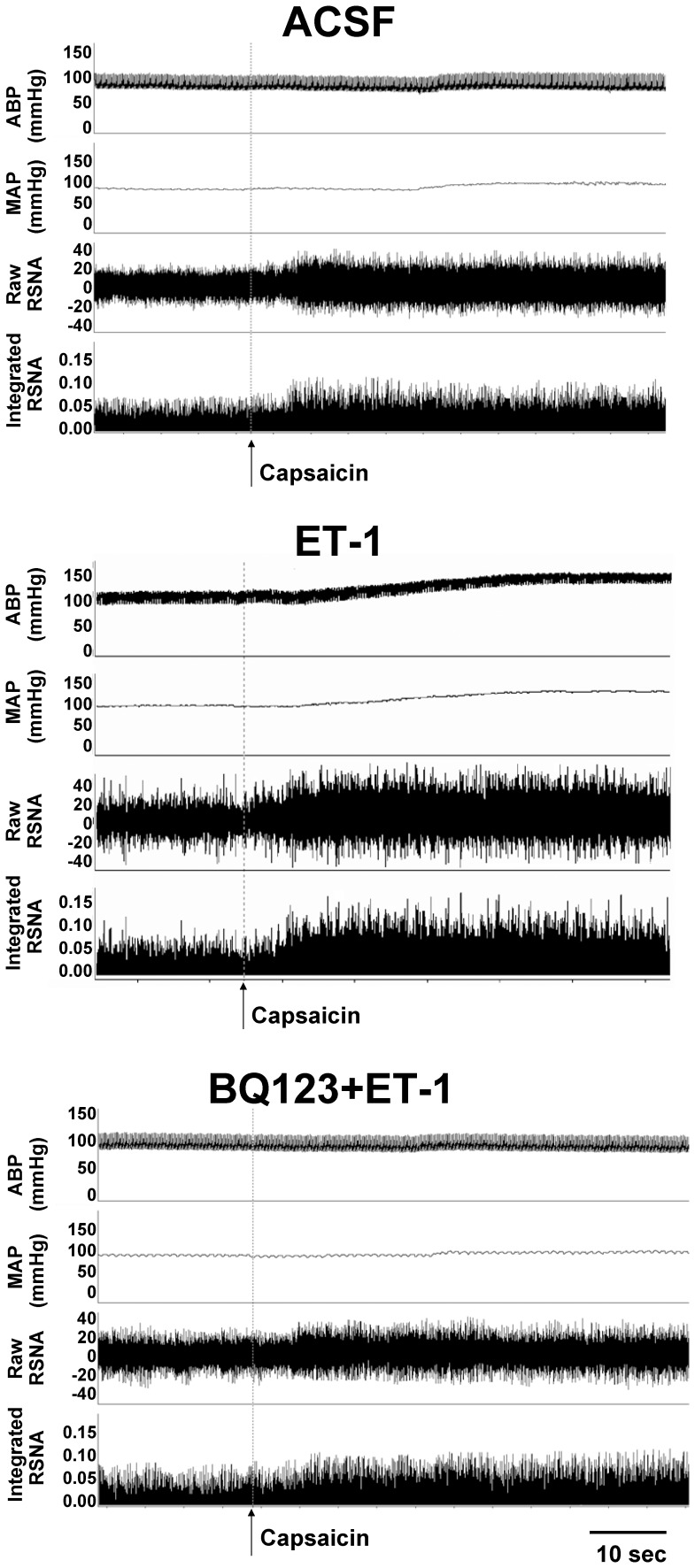
Representative recordings showing the effects of PVN microinjection of ACSF, ET-1 (10 pmol) and ET-1 pretreated with ET_A_ receptor antagonist BQ123 (40 nmol) on CSAR in rats. The CSAR was evaluated by the RSNA and MAP responses to epicardial application of capsaicin.

**Figure 3:**
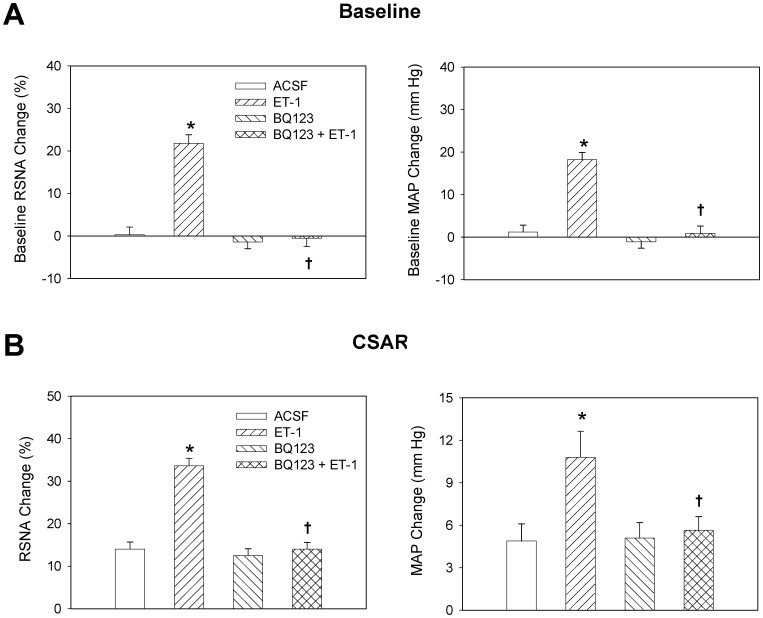
Effects of the PVN microinjection of ACSF, ET-1 (10 pmol), BQ123 (40 nmol) and BQ123+ET-1 on the baseline RSNA and MAP (A) and CSAR (B). The CSAR was evaluated by the RSNA and MAP response to epicardial application of capsaicin. Values are mean±SE. *P<0.05 compared with ACSF, ^†^ *P*<0.05 compared with ET-1 alone. *n = 6* for each group.

The representative recordings showed that PVN microinjection of ET-1 enhanced the CSAR, which was abolished by pretreatment with ET_A_ receptor antagonist BQ123 (Fig. 2). As showing in Figure 3, PVN microinjection of BQ123 alone had no significant effects on the CSAR, RSNA and MAP, but abolished the enhanced CSAR and RSNA responses and pressor response induced by ET-1.

### Effects of Tempol and PEG-SOD

**Figure 4:**
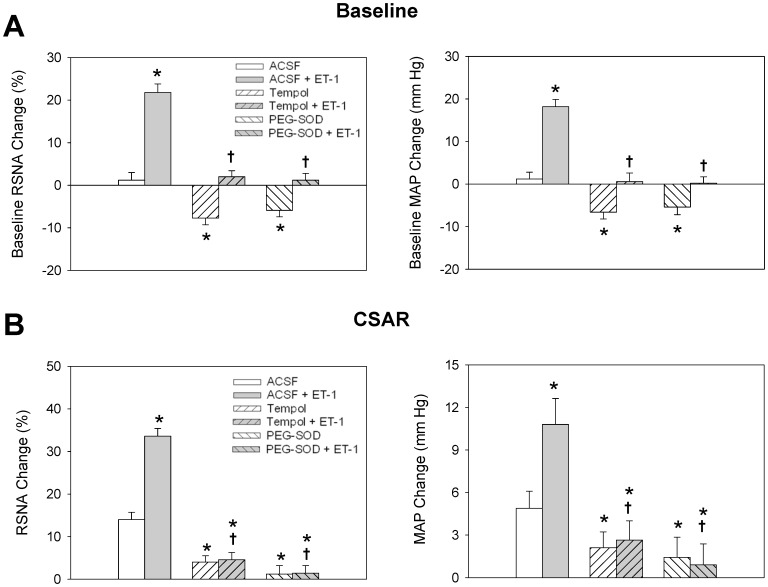
Effects of PVN pretreatment with ACSF, tempol (20 nmol) and PEG-SOD (2 units) on the ET-1 (10 pmol) -evoked baseline RSNA and MAP responses (A) and CSAR responses (B). Values are mean±SE. *P<0.05 compared with ACSF, ^†^ *P*<0.05 compared with ACSF+ET-1. *n = 6* for each group.

Pretreatment with microinjection of superoxide anion scavenger tempol or analogue of endogenous superoxide dismutase PEG-SOD into the PVN inhibited the increasing effects of ET-1 on the CSAR, the baseline RSNA and MAP. Tempol or PEG-SOD alone decreased the CSAR, the baseline RSNA and MAP (Fig. 4).

### Superoxide Anion Level in the PVN

**Figure 5:**
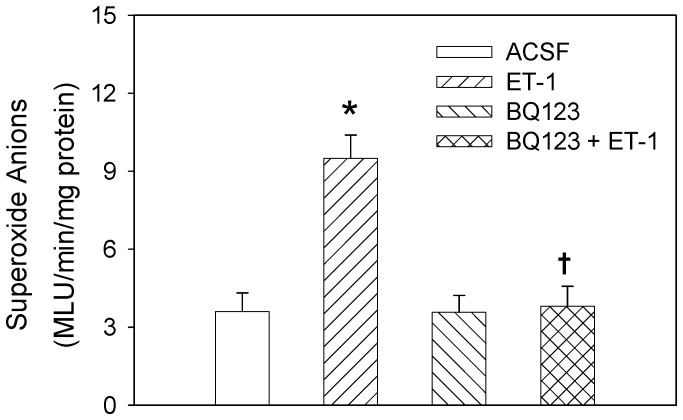
Effects of the PVN microinjection of ACSF, ET-1 (10 pmol), BQ-123 (40 nmol) and BQ123+ET-1 on the superoxide anion level. Values are mean±SE. *P<0.05 compared with ACSF, ^†^ *P*<0.05 compared with ET-1 alone. *n = 6* for each group.

**Figure 6:**
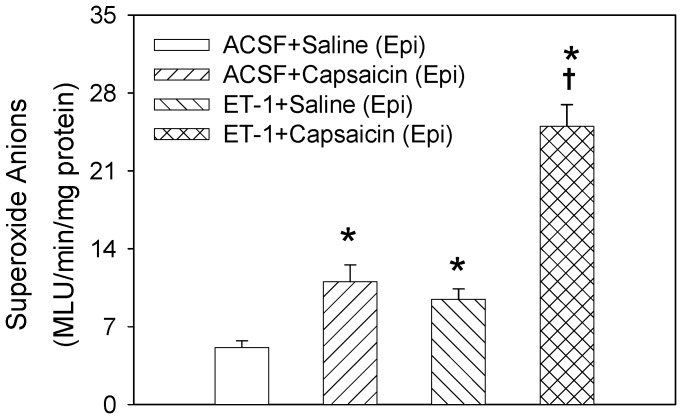
Effects of the PVN pretreatment with ACSF and ET-1(10 pmol) on the response of superoxide anion level to epicardial (Epi) application of saline or capsaicin. Values are mean±SE. *P<0.05 compared with ACSF+Saline, ^†^ *P*<0.05 compared with ACSF+Capsaicin. *n = 6* for each group.

Compared with ACSF, microinjection of ET-1 into the PVN significantly increased the superoxide anion level in the PVN, which was abolished by PVN pretreatment with BQ123. BQ123 alone had no significant effect on the superoxide anion level (Fig. 5). Epicardial application of capsaicin to induce CSAR increased superoxide anion level in the PVN. Pretreatment with PVN microinjection of ET-1 enhanced the response of superoxide anion level to epicardial application of capsaicin (Fig. 6).

## Discussion

Cardiac sympathetic afferent reflex (CSAR) is known as a positive-feedback, sympathoexcitatory cardiovascular reflex. The cardiac sympathetic afferents are stimulated by endogenous chemicals such as bradykinin, adenosine or hydrogen peroxide released from the myocardium during myocardial ischemia and result in sympathetic activation [10]. Direct electrical stimulation of cardiac sympathetic afferent nerves or epicardial application of exogenous chemicals such as bradykinin or capsaicin can induce CSAR [37]. We have found that paraventricular nucleus (PVN) is an important integrative center in the control of the CSAR [38]. The enhanced CSAR in chronic heart failure [12], [39] and hypertension [13] partially contributes to the excessive sympathetic activity that involves in the pathogenesis and the progression of organ damage in these diseases [11], [40], [41].

It has been reported that the aminopropionic acid receptors in the PVN are involved in mediating the pressor response to ET-1 in SFO [21]. The PVN lesion prevents the intracerebroventricular application of ET-1-induced increase in arterial pressure and AVP secretion, showing the importance of the PVN in the effects of ET-1 in the central nervous system [6]. In the present study, microinjection of ET-1 into the PVN enhanced CSAR, increased baseline RSNA and MAP, which was abolished by the pretreatment with ET_A_ receptor antagonist BQ123 in the PVN. However, BQ123 itself in the PVN had no significant effect on the CSAR, RSNA and MAP. These results suggest that exogenous ET-1 in the PVN enhances the CSAR, RSNA and MAP which is mediated by ET_A_ receptors, but endogenous ET-1 in PVN does not involve in the tonic control of the CSAR and sympathetic activity.

Our previous studies have shown that NAD(P)H oxidase-derived superoxide anions in the PVN mediate the enhanced CSAR and sympathetic outflow and are involved in Ang II-induced CSAR-enhancing effects in the PVN in rats with chronic heart failure rats [24] and hypertension [25]. Long-term administration of tempol attenuates ventricular dysfunction and normalizes sympathetic neural control in myocardial infarction rats [42]. It has been reported that the increased ROS level induced by the activation of the ET_A_ receptors in the heart is associated with cardiac hypertrophy in the aryl hydrocarbon receptor null mice [43]. Chronic intravenous ET-1 infusion increases vascular superoxide anions production and plasma thiobarbituric acid-reactive substance (TBARS) level [44]. In the present study, tempol and PEG-SOD were used to scavenge the superoxide anions in the PVN. The PEG-SOD is better in prolonging the circulatory half-life of the native enzymes and enhancing their intracellular access compared with SOD [45]. We found that the PVN microinjection of tempol or PEG-SOD inhibited the excitatory effects of ET-1 on the CSAR, RSNA and MAP. The PVN microinjection of ET-1 increased the superoxide anion level, which was abolished by the pretreatment with ET_A_ receptor antagonist BQ123 in the PVN. Epicardial application of capsaicin to induce CSAR increased superoxide anion level in the PVN which is identical to our previous findings [24], [25]. ET-1 in the PVN further enhanced the response of superoxide anion level to epicardial application of capsaicin. These results suggest that superoxide anions are involved in the modulation of the effects of ET-1 on the CSAR and sympathetic outflow and ET-1 in PVN enhances the CSAR through increasing the generation of superoxide anions. We also found that tempol or PEG-SOD alone in the PVN inhibited the CSAR and decreased the baseline RSNA and MAP, suggesting that the superoxide anions in the PVN play an important role in regulating CSAR and sympathetic activity. However, the blockade of ET_A_ receptors with BQ123 in PVN had no significant effect on the superoxide anion level, suggesting that the endogenous ET-1 in the PVN may be not involved in the tonic generation of superoxide anions in normal rats. It is highly likely that there is some other mechanisms besides ET-1 that may be driving the superoxide anions in PVN with CSAR activation.

In conclusion, exogenous activation of ET_A_ receptors with ET-1 in the PVN enhances the CSAR, increases RSNA and MAP. Superoxide anions in PVN are involved in the effects of ET-1 on the CSAR, RSNA and MAP in rats. However the effects of ET-1 in the PVN in cardiovascular diseases such as hypertension or chronic heart failure remain to be clarified in future experiments.
